# Impacts of the zero mark-up policy on hospitalization expenses of T2DM and cholecystolithiasis inpatients in SC province, western China: an interrupted time series analysis

**DOI:** 10.3389/fpubh.2023.1079655

**Published:** 2023-04-28

**Authors:** Xirui Guo, Yao Xiao, Huan Liu, Qinchuan Li, Qian Jiang, Chun Liu, Fangqing Xie, Hongju Wang, Fang Yang, Xiao Han, Hengbo Yang, Yong Yang, Yongqin Ye, XiaoHong Gan, Enwu Long

**Affiliations:** ^1^Department of Pharmacy, Chengdu Second People's Hospital, Chengdu, China; ^2^College of Optoelectronic Engineering, Chengdu University of Information Technology, Chengdu, China; ^3^Department of Medical, Sichuan Jiaotong Hospital, Chengdu, China; ^4^Department of Pharmacy, School of Medicine, Sichuan Academy of Medical Sciences and Sichuan Provincial People's Hospital, University of Electronic Science and Technology of China, Chengdu, China

**Keywords:** zero mark-up drug policy, policy intervention, interrupted time series, medicine expenses, hospitalization expenses

## Abstract

**Background:**

Since 2009, a series of ambitious health system reforms have been launched in China, including the zero mark-up drug policy (ZMDP); the policy was intended to reduce substantial medicine expenses for patients by abolishing the 15% mark-up on drugs. This study aims to evaluate the impacts of ZMDP on medical expenditures from the perspective of disease burden disparities in western China.

**Method:**

Two typical diseases including Type 2 diabetes mellitus (T2DM) in internal medicine and cholecystolithiasis (CS) in surgery were selected from medical records in a large tertiary level-A hospital in SC Province. The monthly average medical expenses of patients from May 2015 to August 2018 were extracted to construct an interrupted time series (ITS) model to evaluate the impact of policy implementation on the economic burden.

**Results:**

A total of 5,764 cases were enrolled in our study. The medicine expenses for T2DM patients maintained a negative trend both before and after the intervention of ZMDP. It had declined by 74.3 CNY (*P* < 0.001) per month on average in the pre-policy period and subsequently dropped to 704.4 CNY (*P* = 0.028) immediately after the policy. The level change of hospitalization expenses was insignificant (*P* = 0.197), with a reduction of 677.7 CNY after the policy, while the post-policy long-term trend was significantly increased by 97.7 CNY (*P* = 0.035) per month contrasted with the pre-policy period. In addition, the anesthesia expenses of T2DM patients had a significant increase in the level under the impact of the policy. In comparison, the medicine expenses of CS patients significantly decreased by 1,014.2 CNY (*P* < 0.001) after the policy, while the total hospitalization expenses had no significant change in level and slope under the influence of ZMDP. Furthermore, the expenses of surgery and anesthesia for CS patients significantly increased by 320.9 CNY and 331.4 CNY immediately after the policy intervention.

**Conclusion:**

Our study indicated that the ZMDP has been an effective intervention to reduce the excessive medicine expenses for both researched medical and surgical diseases, but failed to show any long-term advantage. Moreover, the policy has no significant impact on relieving the overall hospitalization burden for either condition.

## 1. Introduction

Unreasonably expressing growth in healthcare expenditures has been an increasing matter worldwide. In a range of total healthcare expenditures, pharmaceutical expenditures have been astronomically high in China, accounting for approximately 51.3% of each outpatient visit and 41.3% of each inpatient visit in 2012. These ratios were among the highest contrasted with other countries, whereas the average of OECD was approximately 17% (1). High drug spending contributes as a dominating barrier to healthcare acquisition in China (2). Drug expenditures are exorbitant on account of several reasons, including a specific history of bequeath and profit-driving (3). Triggered by the shortage of government investment and medical revenue, China instated a drug mark-up policy to compensate for hospital expenditures in 1954 (4). The drug mark-up policy refers to drugs sold with a 15–30% profit margin from the wholesale to the retail price (5–7). Statistically, government funds have fallen dramatically from approximately 35% of total hospital revenue in the 1970s to <10% in the 2010s, contributing to medical profits for public hospitals requisite to stay afloat (1). On the other hand, public hospitals afford the dominant medical care in China. However, the revenue from selling medicine to patients instead of offering medical care services accounted for more than 40% of their income since 2000 (8). The primary cause is the excessive utilization of high-price drugs produced by supplier-induced demand (SID) (9, 10). Together with the underpricing of medical services, physicians are keen to prescribe expensive medicine rather than medical services to compensate for their income (11–13). Given the above considerations, the increased drug expenditures immensely overburden patients, resulting in increasing complaints from the public about the affordability of healthcare (7). Therefore, the Chinese government launched the zero mark-up drug policy (ZMDP) to eliminate the 15% mark-up on drugs, alleviating the inflated drug prices (13, 14).

Since 2009, a series of ambitious health system reforms have been launched in China, including universal medical insurance reform, a national essential drug system, development of the primary healthcare system, and pilot reform of public hospitals (6, 15, 16). ZMDP is one of the reforms of public hospitals, and since then, several county-level and urban public hospitals across the country have piloted the policy. By September 2017, all urban public hospitals in China had completed the ZMDP (14, 16, 17). Due to the differences in social and economic development, the distribution of medical resources, the scale of hospitals, and the health level of residents in different regions, the economic burden of patients after ZMDP is also different. Therefore, it is necessary to study the impact of policies on southwest China. The policy reform required public hospitals to reduce 15% of the revenue from drug mark-ups, 70% of which was compensated by adjusting the price of medical services, 20% by implementing financial subsidies, and 10% by exploring hospital potential and enhancing efficiency (5, 18). After the implementation of the policy, public hospitals no longer sold drugs at a mark-up price, which appears to alleviate the drug expenses for patients. However, the policy was accompanied by an increase in medical service charges which reflect labor value, such as treatment, nursing, and surgery, to compensate for inadequate revenues of public hospitals. It follows that the abolition of the drug mark-up policy has resulted in increases and decreases in different expense types. Some studies indicated a decline in drug expenses for chronic diseases, whereas some argued that the policy is not an effective reform to decrease hospitalization expenses in public hospitals (5, 8, 19). It is reported that the extra hospitalization has doubled due to the policy (20). However, there is still no consensus on whether the policy will alleviate the hospitalization expenses of patients.

Furthermore, no article has compared the effects of the ZMDP on medical expenditures for two different diseases from medicine and surgery. As we all know, the medical expense items were various, and the proportions of the expenses were diverse between the two diseases. The reason for selecting the abovementioned two diseases as the research objects is that they are typical diseases in internal medicine and surgery, respectively. In internal medicine, chronic diseases are a major category of diseases; among which, diabetes is the disease with the highest prevalence and has the characteristics of multiple complications, long treatment cycle, and poor prognosis, which has become one of the significant public health problems affecting the social and economic development of the country (21–23). According to International Diabetes Federation, the estimated global prevalence of diabetes aged between 20 and 79 years was 10.5% (537 million people) in 2021; of which, China has the largest number of adults with diabetes at 140 million (24–26). In 2019, the global direct health expenditure on diabetes was approximately USD 760 billion; of which, the spending of China ranked second at an estimated USD 109 billion (27). In surgery, cholecystolithiasis is a disease with an incidence of up to 20%, in which pathogenesis is relatively clear, diagnosis criteria and treatment are standardized, and the curative effect is good, with little difference in the treatment process (28, 29). Selecting two diseases with a large discrepancy in clinical diagnosis, treatment, and medical resource consumption as the research objects is conducive to exploring the effects of ZMDP on the medical burden of patients with different diseases. Therefore, we take a large tertiary level-A hospital as a single center to assess the impact of the policy on patients' medical expenses from the perspective of disease disparities through an ITS analysis.

## 2. Method

### 2.1. Setting and data sources

The data for this study are extracted from the hospital information system (HIS) of a large comprehensive tertiary-grade level-A hospital in Sichuan (SC) province, which had more than 4,300 beds and could be representative of southwest China (30, 31). According to the International Classification of Diseases (ICD-10), the medical diseases were included in the first discharge diagnosis of type 2 diabetes mellitus (T2DM), and the surgical diseases were included in the first discharge diagnosis of cholecystolithiasis (CS). The demographic characteristics, disease information, and medical expenses information of inpatients in 20 months before and after the policy intervention were extracted from medical records. Public hospitals in SC province have fully implemented the reform of abolishing the ZMDP from 20 December 2016. The period from May 2015 to December 2016 was taken as the research series before the policy, and the period from December 2016 to August 2018 was regarded as the research series after the policy.

### 2.2. Quality control

In order to guarantee the quality and quantity of data, the raw data extracted were cleaned, sorted, and logically proofread. The comorbidities associated with the disease were screened and controlled according to the diagnosis and the content of cases, which ensured the same disease was included in patients with similar clinical diagnosis, treatment, surgical operation, and resource consumption. Inpatients whose hospitalization days were <2 days or more than 60 days were excluded. On account of the relatively long-time span of the medical expenses involved in this study, the costs were adjusted according to the medical care category consumer price index (CPI) of SC province in the National Bureau of Statistics (NBS). The CPI of each month in 2016, 2017, and 2018 was adjusted considering the CPI of each month in 2015 as the comparison period, in order to eliminate the influence of inflation. The adjusted CPI is shown in Supplementary Table S1.

### 2.3. Statistical analysis

The general characteristics of patients and the changes in each medical expense before and after the policy implementation were analyzed. Categorical variables were described as counts and percentages, and continuous variables were expressed as means and standard deviations or medians and interquartile range (IOR). Independent group *t*-tests or Mann–Whitney tests were applied to continuous variables, and chi-squared tests were used for categorical variables. All statistical analyses were conducted with SPSS (Statistical Package for the Social Sciences) version 26.0 software, and maps were plotted using Origin 2018.

### 2.4. Interrupted time series analysis

**Figure 1:**
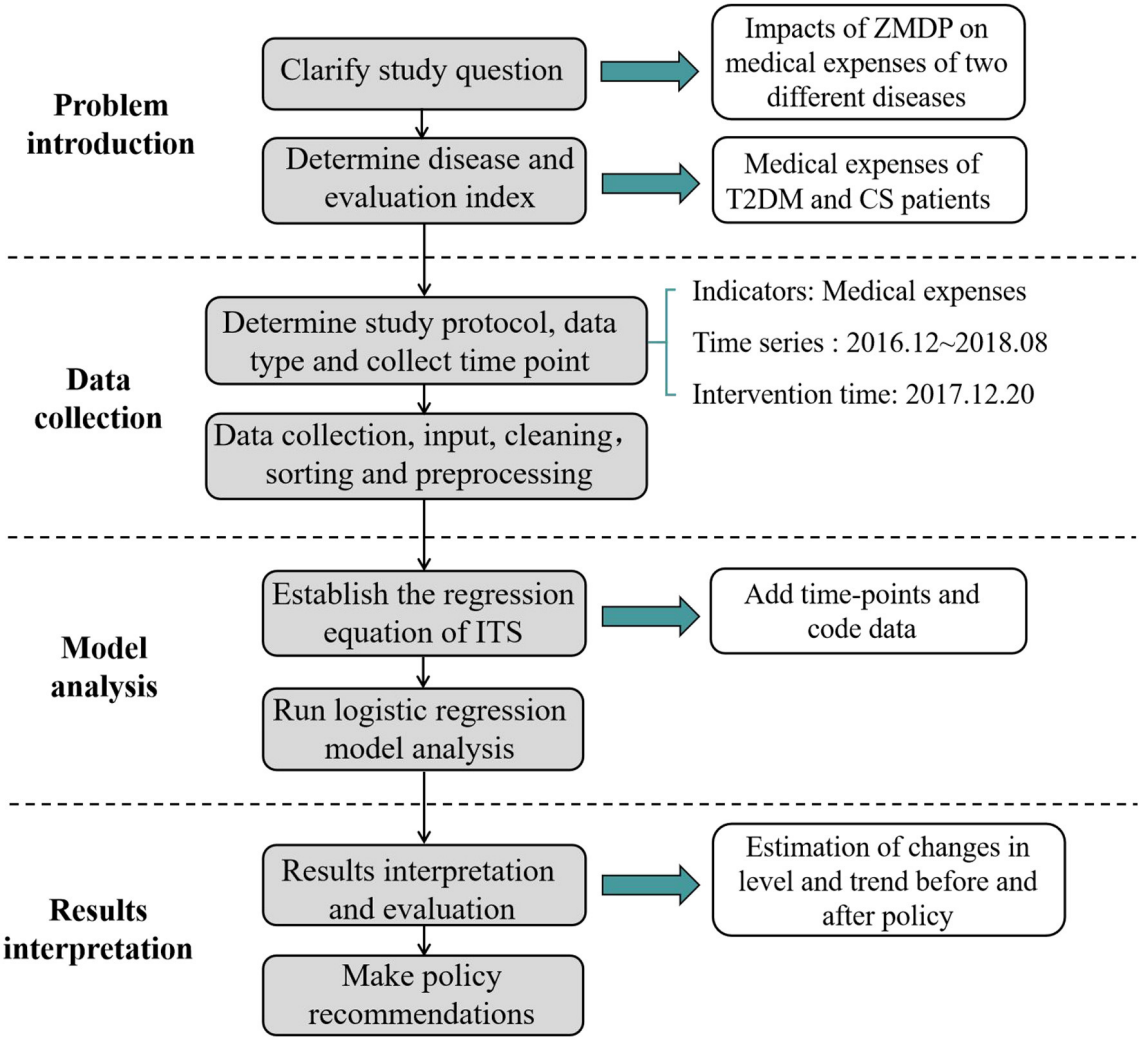
Flowchart of ITS analysis of medical expenses for two diseases.

The ITS analysis is regarded as the strongest quasi-experimental study design to evaluate the longitudinal effect of intervention due to its advantages of quantifying the change in level and trend before and after the intervention, relatively simple analysis operation, and intuitive and clear presentation of results (32, 33). In an interrupted time series (ITS) analysis, data were regularly collected over time at multiple timepoints before and after the intervention to detect whether or not the intervention had a more significant effect than any underlying secular trend (34). In this study, the breakpoint was set on 20 December 2016, when the cancellation of the drug mark-up policy was officially executed as an intervention point of the policy. The data were divided into the pre-policy group (from May 2015 to December 2016) and the post-policy group (from December 2016 to August 2018). Each group had 20 months of data before and after the intervention point. The ITS analysis was conducted with the average medical expenses per patient per time as dependent variables. The following linear regression model is specified to estimate the level and trend in the dependent variable before the policy and the changes in level and trend following the policy. The Durbin–Watson test was used to evaluate the existence of autocorrelations. In this model, the meanings of variables and coefficients are exhibited in Tables 1, 2. The flowchart is shown in Figure 1.


$$\begin{array}{l} Yt=\beta_{0}+\beta_{1}^{*}{\text{time}}_{\text{t}}+\beta_{2}^{*}{\text{intervention}}_{\text{t}}\\ +\;\beta_{3}^{*}{\text{timeafterinterventiont}}_{\text{t}}+e_{\text{t}} \end{array}$$

**Table 1:** Names and interpretation of variables.

**Variable names**	**Interpretation**
**Dependent variables**	
Y_t_	The average of expenses per patient in month *t*
**Independent variables**	
*Time_*t*_*	Time in months at *time_*t*_*from the start of the observation period to the last time point in series
*Intervention_*t*_*	A measure for *time_*t*_*designated as a dummy variable taking the values 0 occurring before intervention and 1 after the intervention (implementation of ZMDP), which was implemented in December 2016 in the series
*Time after interventiont_*t*_*	A continuous variable recording the number of months after the intervention at *time_*t*_*, coded 0 before the policy, and sequentially numbers time periods after the policy

**Table 2:** Names and interpretation of coefficients.

**Coefficient names**	**Interpretation**
β_0_	The baseline level of the average expense at the beginning of the series (i.e., Intercept)
β_1_	The slope of average expense monthly before the intervention of policy (i.e., the baseline trend)
β_2_	The change of average expense in the level immediately after the intervention from the end of the preceding segment
β_3_	The change in the slope from pre- to post-intervention; the sum of β_1_ and β_3_is the post-intervention slope
ε_t_	The random error term unable to be explained by the model

## 3. Results

### 3.1. Characteristics and descriptive analysis results

In our study, 2,922 patients with T2DM were finally enrolled, including 1,445 patients before the policy and 1,477 patients after the policy, respectively. A total of 2,842 patients with CS were included; of which, 1,461 were included before the policy and 1,381 were included after the policy. The basic demographic and clinical characteristics of research objects before and after implementing the ZMDP are displayed in Table 3. Compared with the pre-policy period, there was no significant difference (*P* > 0.05) in gender, age, and the number of complications in patients with T2DM and CS after the policy, indicating that the baselines of patients before and after the policy implementation were consistent. The average hospitalization days differed significantly (*P* < 0.001) between the two groups before and after the policy in the two diseases, reflecting the improvement in the utilization of medical resources and the work efficiency of medical staff after the reform.

**Table 3:** Demographic and clinical characteristics of patients included before and after the reform.

**Characteristics**	**T2DM/No. (%)**			**CS/No. (%)**		
	**Pre-policy (*****N** =* **1,445)**	**Post-policy (*****N** =* **1,477)**	***P*** **-value**	**Pre-policy (*****N** =* **1,461)**	**Post-policy (*****N** =* **1,381)**	***P*** **-value**
**Gender**						
Male	848 (58.7)	871 (59.0)	0.886	999 (32.4)	924 (33.4)	0.591
Female	597 (41.3)	606 (41.0)		462 (67.6)	457 (66.6)	
**Age**						
$\bar{x}$ ± s	61.4 ± 13.9	60.9 ± 14.2	0.332	48.4 ± 14.5	48.3 ± 14.4	0.889
<40	97 (6.7)	107 (7.3)	0.800	438 (30.0)	416 (30.1)	0.977
40−59	499 (34.6)	528 (35.7)		671 (45.9)	622 (45.1)	
60−79	727 (50.3)	722 (48.9)		337 (23.1)	326 (23.6)	
≥80	122 (8.4)	120 (8.1)		15 (1)	17 (1.2)	
**Average hospitalization days**						
$\bar{x}$ ± s	13.3 ± 4.3	12.6 ± 3.8	< 0.001^*^	7.4 ± 2.9	6.6 ± 2.6	< 0.001^*^
**Payment type**						
Individual payment	414 (28.7)	324 (21.9)	< 0.001^*^	431 (29.5)	368 (26.6)	0.072
Medicare payment	1,031 (71.3)	1,153 (78.1)		1,030 (70.5)	1,013 (73.4)	
**Number of complications**						
0	12 (0.8)	8 (0.5)	0.080	1,135 (77.8)	1,021 (73.9	0.120
1−2	174 (12.1)	145 (9.8)		287 (19.6)	315 (22.8)	
3−6	720 (49.8)	737 (49.9)		37 (2.5)	41 (3)	
7−9	346 (23.9)	357 (24.2)		2 (0.1)	4 (0.3)	
≥10	193 (13.4)	230 (15.6)		0 (0)	0 (0)	

T2DM, Type 2 diabetes mellitus; CS, cholecystolithiasis; No., number; %, percentage; $\bar{x}$, means; s, standard deviations.

* A statistically significant difference.

### 3.2. Comparative analysis of hospitalization expenses

**Figure 2:**
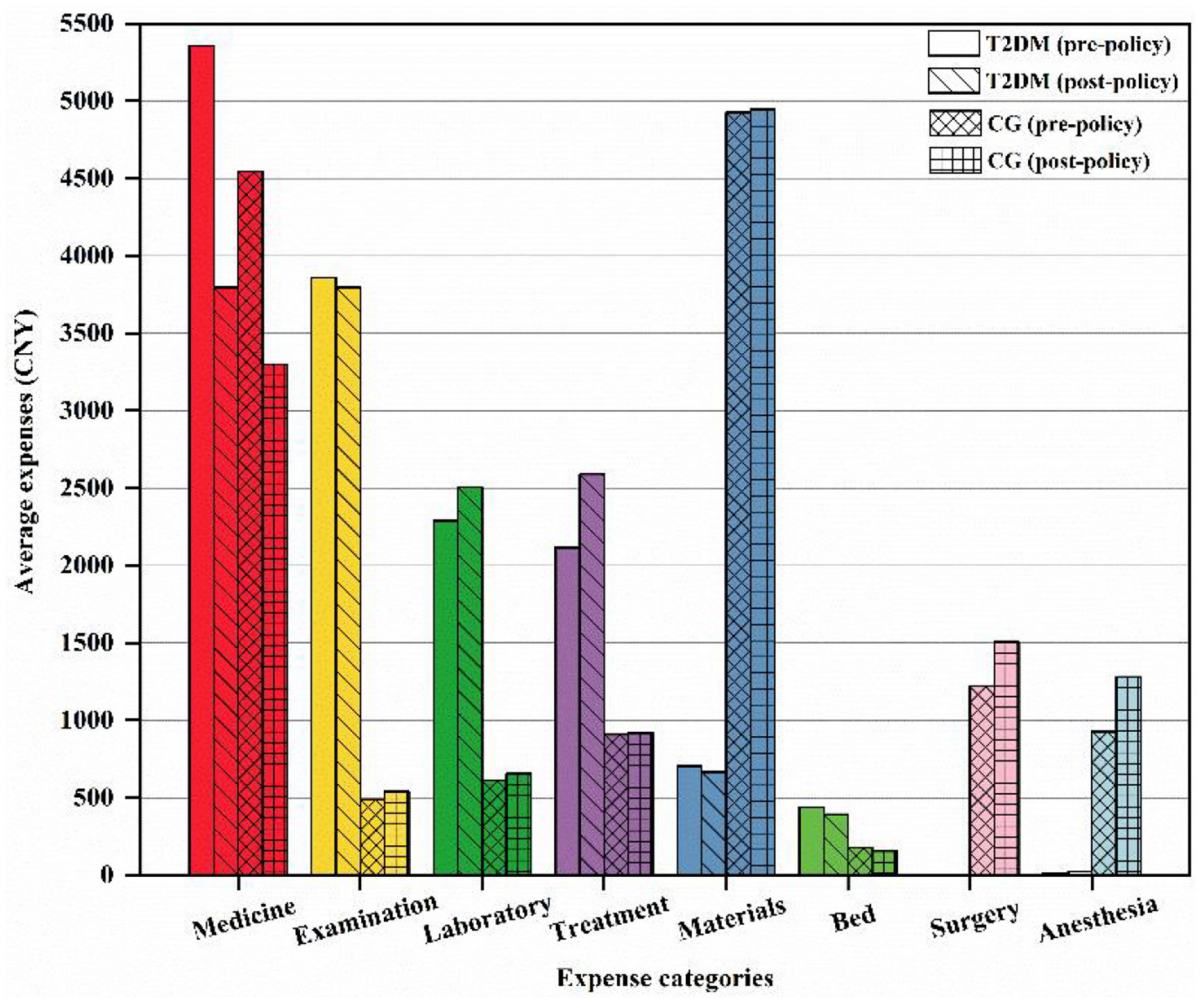
Comparison of average expenses for two diseases before and after the policy.

Figure 2 shows the comparison of expense categories, including medicine expenses, examination expenses, laboratory expenses, treatment expenses, materials expenses, bed expenses, surgery expenses, and anesthesia expenses, between the two diseases before and after the implementation of ZMDP. For the medical illness T2DM, the expenses of medicine, examination, laboratory, and treatment ranked in the top four among the various expenses. Conversely, the expenses of materials were the highest among various hospitalization expenses for surgical disease CS, followed by the expenses of medicine, surgery, anesthesia, etc. Due to the positively skewed distribution of the expenses, data were presented as a median and interquartile range, and Mann–Whitney tests were applied to comparative analysis, as shown in Table 4. We found that the expenses of medicine, treatment, materials, bed, and total hospitalization for T2DM patients reduced significantly (*p* < 0.001) after the implementation of ZMDP, of which medicine expenses decreased by 1,394.7 CNY, and the total hospitalization expenses declined by 1,194.1 CNY. In contrast, the expenses of laboratory and treatment increased significantly (*p* < 0.001) in the post-policy period, rising by 145.2 CNY and 367.7 CNY, separately. For CG patients, the expenses of medicine, bed, and total hospitalization dropped significantly (*p* < 0.001) after the policy, of which medicine expenses decreased by 1,079.7 CNY, and total hospitalization expenses declined by 568.0 CNY. On the contrary, the expenses of surgery and anesthesia increased significantly (p < 0.001) after the policy, increasing by 285.5 CNY and 298.5 CNY, respectively.

**Table 4:** Comparative analysis of the medical expenses for two diseases before and after the reform.

**Items**	**T2DM**			**CS**		
	**Pre-reform (*****N** =* **1,461)**	**Post-reform (*****N** =* **1,381)**	***P*** **value**	**Pre-reform (*****N** =* **1,445)**	**Post-reform (*****N** =* **1,477)**	***P*** **value**
Medicine	4,570.3 (3,606.2)	3,175.6 (2,417.9)	< 0.001^*^	4,255.2 (2,210.8)	3,017.0 (1,858.7)	<0.001^*^
Examination	3,632.3 (1,747.1)	3,639.5 (1,655.2)	0.381	271.0 (254.1)	269.8 (243.8)	0.002^*^
Laboratory	1,979.6 (1,121.0)	2,124.8 (1,161.6)	<0.001^*^	505.0 (226.0)	578.2 (317.2)	0.045^*^
Treatment	1,729.6 (1,074.9)	2097.3 (1387.7)	<0.001^*^	908.0 (325.5)	859.8 (312.6)	0.432
Materials	499.8 (479.6)	426.30 (445.9)	<0.001^*^	4,988.9 (1,235.2)	4,960.6 (14,02.0)	0.352
Bed	416.0 (219.3)	364.0 (176.1)	<0.001^*^	159.3 (93.9)	145.8 (78.2)	<0.001^*^
Surgery	0.0 (0.0)	0.0 (0.0)	0.502	1,207.9 (20.4)	1,493.4 (21.0)	<0.001^*^
Anesthesia	0.0 (0.0)	0.0 (0.0)	0.076	959.8 (100.0)	1258.3 (124.4)	<0.001^*^
Total	13,411.9 (6,998.8)	12,217.9 (5,978.8)	<0.001^*^	13,468.73 (3,610.3)	12,900.75 (3,566.9)	<0.001^*^

N, number; T2DM, Type 2 diabetes mellitus; CS, cholecystolithiasis.

* A statistically significant difference.

### 3.3. The ITS analysis of hospitalization expenses

In the ITS analysis, 2,922 patients with T2DM and 2,842 patients with CS across 40 months from May 2015 to August 2018 were included. Overall, the average medicine expenses for T2DM patients before the policy intervention was 5354.5 CNY, and it was higher than that after the policy, which was 3795.2 CNY. The average total hospitalization expenses before and after the policy were 14,778.7 CNY and 13,767.8 CNY, respectively. Among the CS patients, the average medicine expenses were 4,543.5 CNY in the pre-intervention period and 3299.4 CNY in the post-intervention period. The average total hospitalization expenses were 13,807.0 CNY and 13,303.5 CNY before and after the policy, separately.

#### 3.3.1. The ITS analysis of monthly average hospitalization expenses for T2DM patients

**Figure 3:**
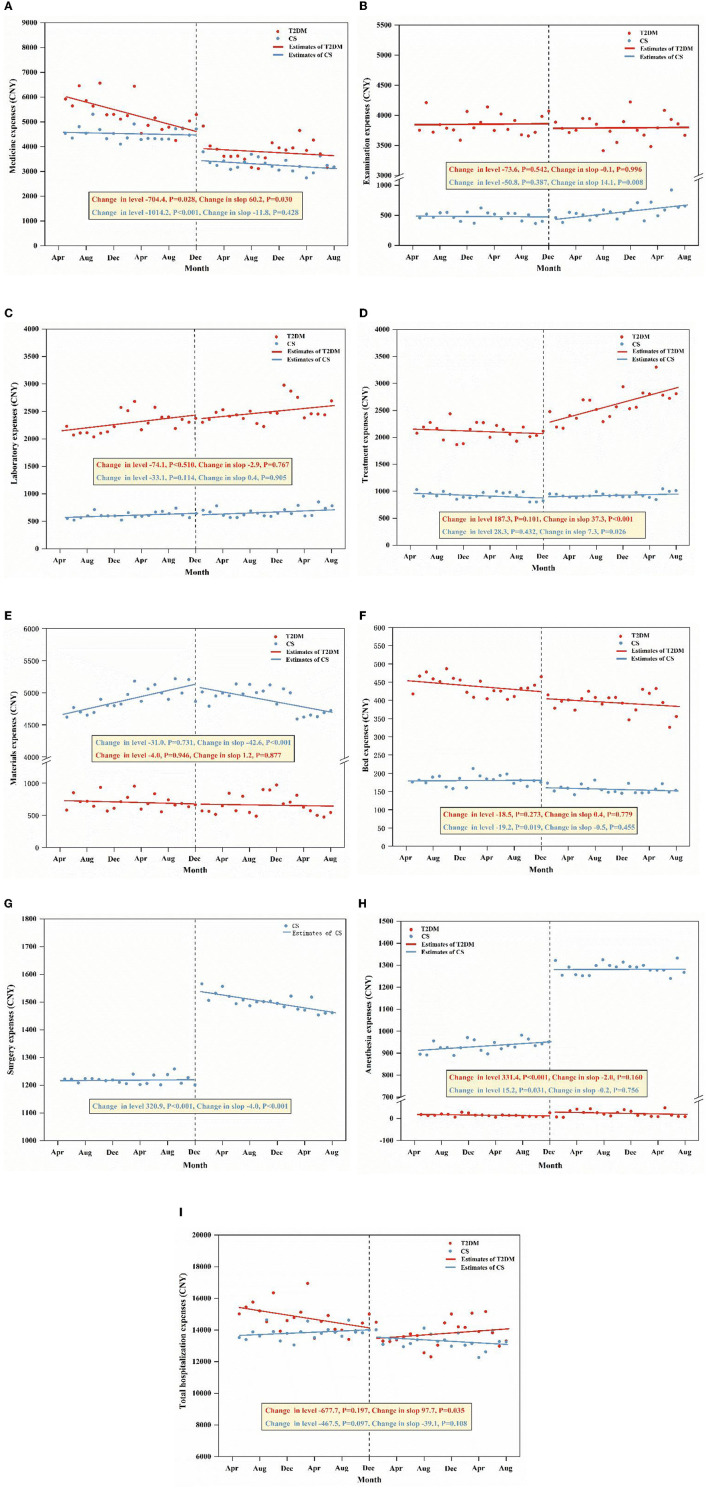
ITS analysis of monthly average medicine expenses **(A)**, examination expenses **(B)**, laboratory expenses **(C)**, treatment expenses **(D)**, materials expenses **(E)**, bed expenses **(F)**, surgery expenses **(G)**, anesthesia expenses **(H)**, and total hospitalization expenses **(I)** for two diseases.

The ITS analysis showed that the medicine expenses for T2DM patients expressed a statistically significant declining baseline trend before the ZMDP was implemented, as exhibited in Figure 3, with an average monthly decrease of 74.3 CNY (*P* < 0.001). The total hospitalization expenses displayed a significant downward baseline slope in the expenses of 67.5 CNY (*P* = 0.039) per month before the policy. In the first month after the policy intervention, the medicine expenses dropped significantly by 704.4 CNY (*P* = 0.028), implying that the implementation of the policy exerted an immediate negative level change in medicine expenses. Although the total hospitalization expenses declined immediately by 677.7 CNY (*P* = 0.197), the slope change was not statistically significant. After the policy intervention, the trend of medicine expenses increased by 60.2 CNY compared with the trend before the policy, which was a significant slope change (*P* = 0.030). In other words, the long-term trend of medicine expenses maintained negative but almost flat, with a monthly reduction of 14.1 CNY (β_1_ + β_3_). The long-term trend of total hospitalization changed from downward to upward, with a monthly increase of 30.2 CNY (β_1_ + β_3_), which was a significant change in the post-policy period with respect to the pre-policy period (P = 0.035). Moreover, the expense of treatment for T2DM patients changed from a downward trend to an upward trend, with a monthly increase of 32.7 CNY (β_1_ + β_3_), and this change in slope was statistically significant (*P* < 0.001). The anesthesia expenses of T2DM patients immediately increased by 15.2 CNY after the policy intervention, and this level change was significant (P = 0.031). Apart from that, there was no significant change in the level and slope for other monthly average expenses before and after the intervention of the ZMDP. The diagrams of the ITS analysis are shown in Figure 3. Detailed model parameters are displayed in Table 5.

**Table 5:** Model validation and parameter estimation of average medical expenses for two diseases.

**Items**	**Intercept (**β_**0**_**)**		**Time (**β_**1**_**)**		**Intervention (**β_**2**_**) (change in level)**		**Time after Intervention (**β_**3**_**) (change in slope)**		**AC check**
	**Es** β_0_	***P*** **-value**	**Es** β_1_	***P*** **-value**	**Es** β_2_	***P*** **-value**	**Es** β_3_	***P*** **-value**	**D-value**
**T2DM**									
Medicine	6,120.9	<0.001^*^	−74.3	<0.001^*^	−704.4	0.028^*^	60.2	0.030^*^	1.560
Examination	3,845.1	<0.001^*^	0.8	0.912	−73.6	0.542	−0.1	0.996	1.935
Laboratory	2,130.0	<0.001^*^	15.3	0.031^*^	−74.1	0.510	−2.9	0.767	1.211
Treatment	2,159.9	<0.001^*^	−4.6	0.509	187.3	0.101	37.3	<0.001^*^	2.052
Materials	731.9	<0.001^*^	−2.7	0.625	−4.0	0.946	1.2	0.877	1.742
Bed	456.7	<0.001^*^	−1.6	0.123	−18.5	0.273	0.4	0.779	1.310
Anesthesia	18.2	<0.001^*^	−0.3	0.492	15.2	0.031^*^	−0.2	0.756	1.886
Toal	15,483.3	<0.001^*^	−67.5	0.039^*^	−677.7	0.197	97.7	0.035^*^	1.762
**CG**									
Medicine	4,593.0	<0.001^*^	−5.5	0.601	−1,014.2	<0.001^*^	−11.8	0.428	1.762
Examination	504.9	<0.001^*^	−1.4	0.703	−50.8	0.387	14.1	0.008^*^	2.653
Laboratory	567.2	<0.001^*^	4.3	0.115	−33.1	0.445	0.5	0.905	1.862
Treatment	963.4	<0.001^*^	−4.8	0.040^*^	28.3	0.432	7.3	0.026^*^	1.591
Materials	4,666.0	<0.001^*^	23.2	<0.001^*^	−31.0	0.731	−42.6	<0.001^*^	1.696
Bed	179.8	<0.001^*^	0.1	0.885	−19.2	0.019	−0.5	0.455	2.209
Surgery	1,217.6	<0.001^*^	0.2	0.828	320.9	<0.001^*^	−4.0	<0.001^*^	2.617
Anesthesia	905.8	<0.001^*^	2.1	0.040^*^	331.4	<0.001^*^	−2.0	0.160	2.013
Toal	13,597.7	<0.001^*^	18.2	0.285	−467.5	0.097	−39.1	0.108	2.093

Regression coefficients, standard errors, P-values, and autocorrelations from the multiple regression analysis type of segmented regression analysis of interrupted time series for models (average medical expenses as the dependent variable).

Es, estimated; AC, autocorrelation check; D-Value, Durbin Watson statistic; T2DM, Type 2 diabetes mellitus; CS, cholecystolithiasis.

* A statistically significant difference.

#### 3.3.2. The ITS analysis of monthly average hospitalization expenses for CS patients

In the mode for the medicine expenses of CS patients, the trend before the ZMDP implementation was negative and did not exhibit statistical significance (*P* = 0.061), which implies that the change of medicine expenses per month had no variation in the pre-policy period. The total hospitalization expenses displayed no significant (*P* = 0.285) change per month before the policy was implemented. Meanwhile, the expenses of materials and anesthesia showed a statistically notable increasing baseline trend in expenses of 23.2 CNY (*P* < 0.001) and 2.1 CNY (*P* = 0.040) per month before the policy. In addition, there was a significantly increasing baseline (*P* = 0.040) in the treatment expenses of 4.8 CNY per month before the policy. In the first month after the intervention, the medicine expenses dropped by 1014.2 CNY immediately, and this level change was statistically significant (*P* < 0.001). This demonstrates that the policy exerted a significant level decrease in medicine expenses. The total hospitalization expenses dropped by 467.5 CNY immediately and this level change was not significant (*P* = 0.097), indicating that the policy had no immediate effect on reducing the total hospitalization expenses. Moreover, the expenses of surgery and anesthesia for CS patients increased immediately by 320.9 CNY and 331.4 CNY in level after the policy, and these were statistically significant (*P* < 0.001). After the policy intervention, the trend of medicine expenses remained negative, with a monthly decrease of 17.3 CNY (β_1_ + β_3_), and this decrease in slope was not significant (*P* = 0.428) contrasted with the trend in the pre-policy period. Similarly, the change from upward to downward in the total hospitalization expenses after the policy was insignificant (*P* = 0.108). In addition, the trend in the expense of surgery changed from upward to downward and declined by 3.8 CNY (β_1_ + β_3_) per month before and after the policy, and the change in slope was significant (*P* < 0.001). The trend of examination expenses and treatment expenses in the post-policy period began to be positive while that of materials expenses and surgery expenses turned negative. These changes in slope were all statistically significant, indicating that the policy exerted long-term slope change in the expenses of CS patients. The diagrams of ITS analysis are shown in Figure 3. Detailed model parameters are displayed in Table 5.

Meanwhile, the ITS analysis was also conducted on the percentage of each expense in order to demonstrate the changing composition of total expenses, and the results are shown in Supplementary Figure S1. For T2DM patients, in the first month after the reform, the proportion of medicine expenses decreased by 3.869% (*P* = 0.007), indicating that the immediate effect of policy intervention was significant (*p* < 0.05). After the reform, the proportion of medicine expenses still maintained a downward trend, indicating that the effect of the reform was long-term sustainability (*p* < 0.05). For CS patients, before the intervention, the percentage of drug costs and treatment costs showed a significant downward trend, and the proportion of material costs showed a significant upward trend (*P* < 0.05). In the first month after the reform intervention, the proportion of medicine expenses and examination expenses decreased by 6.004% and 0.367%, respectively, which indicates that the immediate effect of policy intervention was significant (*P* < 0.05) (Supplementary Table S2).

## 4. Discussions

### 4.1. Interpretation of findings

Our study found that the immediate reductions in medicine expenses were as intended under the function of ZMDP, which is consistent with previous studies showing the policy can observably attenuate the burden of expenses for medicine (5, 18, 19). Wang et al. (5) indicated that the ZMDP could curb the increase in medicine and hospitalization expenses for inpatients with COPD. Du et al. (19) showed that ZMDP can reduce the drug cost for chronic disease outpatients in the tertiary hospital and their economic burden. However, the overall hospitalization expenditures were found to be declined insignificantly after the policy for both diseases, which is inconsistent with the results of previous studies (5, 19). In terms of long-term trend change, the medicine expenses of the researched two diseases continued to decline after the policy, but the decrease was insignificant. Furthermore, the long-term decrease in hospitalization expenses was not satisfactory and even rebounded for T2DM patients. These revealed that the policy had no long-term impact to a certain extent, so follow-up policies are required for continuous improvement, such as the Chinese national centralized drug procurement of drugs and the medical insurance negotiation of drug prices (35–38).

Although drug expenses for the two diseases have fallen, different medical service expenses are increasing for patients with different diseases. Our results verified that the treatment expenses for T2DM patients have increased dramatically after the policy, which may be the dominant reason why the total hospitalization expenses have soared after the policy. Moreover, the expenses of surgery and anesthesia for patients with CS also have risen markedly after the implementation of the policy, and the expenses of examination and treatment continued to grow under the influence of the policy. These results revealed that the percentage of drug expenses in total hospitalization expenses had indeed reduced, while the medical service expenses reflecting the labor value of medical staff and the overall hospitalization expenditure are still increasing. Consequently, the disparities in healthcare expenditure among diseases might be aggravated by the impact of the ZMDP, which implies it is one-sided to analyze the impact of ZMDP on the economic burden of patients only from a single disease or multiple diseases of the same type. Meanwhile, accessibility to medical services and the huge economic profits that follow may promote doctors to provide more services, along with the risk of resource waste. Therefore, subsequent policies are required to diminish healthcare discrepancies further, mitigate the medical burden on patients, ensure the financial stability of public hospitals, and advance healthcare reform (7, 35, 36).

### 4.2. Strengths and limitations

This article discussed the influence of China's healthcare reform of ZMDP from the angle of patients' medical burden in SC province through the ITS analysis. The segmented regression analysis of the ITS model is appropriate for studying the effects of interventions in changing results, immediately and over time. The advantage of ITS is that it can control and eliminate the influence of other factors on the long-term trend change caused by the observation index, and it can correctly assess the real impact of the intervention on the outcomes and exhibit intuitive graphics of the results (39, 40). Therefore, ITS is suitable for the evaluation of intervention effects in the field of medical and health research. Moreover, this study first proposed to evaluate the impact of ZMDP from the perspective of two different types of diseases in internal medicine and surgery, which was not mentioned in previous studies. Medicine costs dominate for patients with medical diseases mainly treated with drugs, while the expenses of surgery, anesthesia, or materials costs, account for a relatively high proportion of patients with surgical diseases. Apparently, abolishing mark-up on drug prices may exert a different impact on patients with different diseases. Hence, our study comprehensively evaluates the impact of ZMDP on the economic burden of patients from the perspective of disease burden disparities, which conduce to reduce the disparities of disease burden, avoiding broad-brush reform. More importantly, evaluating the impact of the policy reform can provide decision-making references for the policy formulation of the health system reform, which is of practical significance to tackling the problem of health disparities and avoiding the one-size-fits-all approach (7, 41). The degree of influence varies with different diseases. Due to the limitations of data sources and data volume, this study has not done empirical research on other diseases and did not consider the impact of different insurance programs on the total hospitalization expenses. In addition, this study only analyzes the data from a single center in Sichuan Province. If the data from more urban public hospitals in Sichuan Province can be covered, the results will have more reference value and practical significance.

### 4.3. Policy implications

The reform of ZMDP has had mixed success as the medicine expenses were reduced as expected for both T2DM and CS patients, while the total hospitalization burden for T2DM patients sustained to rise after the policy. Our findings suggest that the following policies are required to avoid a one-size-fits-all approach and pay more attention to the rise in the expenses of examination, surgery, and anesthesia for surgical diseases, such as CS, and the increase in treatment expenses of medical illnesses, such as T2DM, to control the growth of total hospitalization expenditures. The subsequent reforms need to focus the cost control on reducing hospitalization expenses for each different disease, such as the trial of Diagnosis Related Groups (DRGS) payment, which seems to be more accurately executed to patients and can also improve the enthusiasm and initiative of medical service providers in charge control (42). Furthermore, this study may contribute to providing empirical support to the evaluation and improvement of subsequent policy effects, help refine the regional policies, and promote follow-up reform in the western region of China. It may also conduce to underdeveloped countries that intend to relieve their citizens' medical burdens.

## Data availability statement

The original contributions presented in the study are included in the article/Supplementary material, further inquiries can be directed to the corresponding authors.

## Author contributions

XGu designed the study, determined the structure of the paper, and wrote the manuscript. XGu, CL, FY, QL, and QJ conducted background research and policy interpretation. XGu, XH, HW, and FX were responsible for the collection, arrangement, and cleaning of raw data. XGu, YX, HL, and HY performed statistics and plots. YYa, YYe, XGu, and EL supervised the study and revised the paper. All authors reviewed and approved the manuscript.
