# Supplementary material for: Impacts of the zero mark-up policy on hospitalization expenses of T2DM and cholecystolithiasis inpatients in SC province, western China: an interrupted time series analysis
Source: Front Public Health. 2023 Apr 28;11:1079655. doi: 10.3389/fpubh.2023.1079655 (PMC10177657; doi:10.3389/fpubh.2023.1079655)
Supplement: Supplementary file 3 [file Table_3.docx]

Supplementary Material

Table S1. Adjusted CPI for each time point（the same month in 2015=100）

| Year | Month | CPI | Year | Month | CPI | Year | Month | CPI |
| --- | --- | --- | --- | --- | --- | --- | --- | --- |
| 2016 | 1 | 100.8 | 2017 | 1 | 105.0 | 2018 | 1 | 107.1 |
| 2016 | 2 | 100.8 | 2017 | 2 | 105.5 | 2018 | 2 | 107.3 |
| 2016 | 3 | 101.0 | 2017 | 3 | 105.6 | 2018 | 3 | 107.7 |
| 2016 | 4 | 101.1 | 2017 | 4 | 105.6 | 2018 | 4 | 107.9 |
| 2016 | 5 | 101.2 | 2017 | 5 | 105.8 | 2018 | 5 | 108.8 |
| 2016 | 6 | 101.4 | 2017 | 6 | 106.0 | 2018 | 6 | 1090 |
| 2016 | 7 | 101.5 | 2017 | 7 | 106.1 | 2018 | 7 | 109.1 |
| 2016 | 8 | 101.7 | 2017 | 8 | 106.1 | 2018 | 8 | 109.4 |
| 2016 | 9 | 101.8 | 2017 | 9 | 106.2 |  |  |  |
| 2016 | 10 | 102.1 | 2017 | 10 | 106.2 |  |  |  |
| 2016 | 11 | 102.8 | 2017 | 11 | 106.2 |  |  |  |
| 2016 | 12 | 103.3 | 2017 | 12 | 106.2 |  |  |  |

**Table S2.** Model validation and parameter estimation of medical expenses percentage for two diseases

| Items | Intercept (β_0_) | | Time(β_1_) | | Intervention (β_2_)  (Change in level) | | Time After Intervention (β_3_)  (Change in slope) | | AC Check |
| --- | --- | --- | --- | --- | --- | --- | --- | --- | --- |
|  | Es β_0_ | P-value | Es β_1_ | P-value | Es β_2_ | P-value | Es β_3_ | P-value | D-value |
| T2DM |  |  |  |  |  |  |  |  |  |
| Medicine | 38.34 | <0.001^*^ | -0.37 | <0.001^*^ | -3.87 | 0.007^*^ | 0.24 | 0.016^*^ | 1.036 |
| Examination | 26.25 | <0.001^*^ | 0.13 | 0.010^*^ | 0.73 | 0.353 | -0.18 | 0.012^*^ | 2.337 |
| Laboratory | 14.24 | <0.001^*^ | 0.19 | <0.001^*^ | 0.36 | 0.621 | -0.18 | 0.007^*^ | 1.579 |
| Treatment | 13.56 | <0.001^*^ | 0.04 | 0.228 | 2.05 | <0.001^*^ | 0.1 | 0.005^*^ | 1.196 |
| Materials | 4.26 | <0.001^*^ | 0.01 | 0.663 | -0.17 | 0.600 | -0.02 | 0.581 | 1.594 |
| CG |  |  |  |  |  |  |  |  |  |
| Medicine | 32.80 | <0.001^*^ | -0.12 | 0.010^*^ | -6.00 | <0.001^*^ | 0.05 | 0.423 | 1.481 |
| Examination | 3.50 | <0.001^*^ | -0.02 | 0.266 | -0.37 | 0.197^*^ | 0.12 | <0.001^*^ | 2.542 |
| Laboratory | 4.30 | <0.001^*^ | 0.02 | 0.136 | -0.14 | 0.417 | 0.01 | 0.529 | 1.401 |
| Treatment | 7.10 | <0.001^*^ | -0.04 | <0.001^*^ | 0.46 | 0.022^*^ | 0.07 | <0.001^*^ | 1.521 |
| Materials | 34.77 | <0.001^*^ | 0.14 | 0.002^*^ | 0.69 | 0.318 | -0.23 | <0.001^*^ | 1.555 |
| Surgery | 9.36 | <0.001^*^ | 0.01 | 0.731 | 2.76 | <0.001^*^ | -0.01 | 0.475 | 1.87 |
| Anesthesia | 6.86 | <0.001^*^ | 0.02 | 0.064 | 2.73 | <0.001^*^ | -0.02 | 0.308 | 2.381 |

Regression coefficients, standard errors, P-values, and autocorrelations from the multiple regression analysis type of segmented regression analysis of interrupted time series for models (expenses percentage as the dependent variable).

Abbreviations: Es, estimated; AC, Autocorrelation Check; D-Value, Durbin Watson statistic; T2DM, Type 2 diabetes mellitus; CS, cholecystolithiasis.

* refers to statistically significant difference.

| A  | B  |
| --- | --- |
| C  | D  |
| E  | F  |
| Figure S1. ITS analysis of the percentage of medicine expenses(A), examination expenses(B), laboratory expenses(C), treatment expenses(D), materials expenses(E), and surgery and anesthesia expenses(F) for two diseases. | |
